# Genomic Insights into the Fish-Pathogenic Mycobacterium pseudoshottsii Strain AR Recovered from Meagre (*Argyrosomus regius*)

**DOI:** 10.1128/MRA.01099-20

**Published:** 2020-11-19

**Authors:** Panagiota Stathopoulou, Elias Asimakis, Yiannis Petropoulos, Georgia Apostolopoulou, George Tsiamis

**Affiliations:** a Department of Environmental Engineering, University of Patras, Agrinio, Greece; University of Maryland School of Medicine

## Abstract

Mycobacteriosis can cause morbidity and mortality and is characterized as a disease with zoonotic potential that is found worldwide in both fresh and marine fish. Here, we report the draft genome sequence of Mycobacterium pseudoshottsii strain AR, an isolate recovered from infected meagre (Argyrosomus regius) raised on farms in Greece.

## ANNOUNCEMENT

The meagre (Argyrosomus regius) is considered to be one of the best potential candidates for large‐scale fish farming in the Mediterranean (1). Although global meagre production is reported to have increased rapidly, one of the most important bottlenecks remains systemic granulomatosis (2). The etiology of the disease has been related to nocardiosis in one incident from Greece (3), while two cases of mycobacteriosis have been reported from Turkey (4, 5). Here, we report on the genomic analysis of a granuloma-associated *Mycobacterium* strain isolated from infected meagre raised on farms in Greece.

In late 2019, meagre showing lethargy, anorexia, abdominal swimming, and enlarged internal organs were recorded in fish farms. Pathogen isolation was attempted, with the affected spleens exhibiting white nodules. The tissues were aseptically dissected, decontaminated, homogenized (6), and inoculated onto Middlebrook 7H10 agar (7). Incubation was performed at 30°C for 2 months. The first isolate grew 6 weeks later. The colonies were rough, not emulsifiable in water, and unable to grow at 37°C or below 20°C.

Genomic DNA was extracted from a single colony using lysis buffer containing lysozyme (Sigma-Aldrich), according to Haught et al. (8). DNA was quantified in triplicates with the Quant-iT double-stranded DNA assay (Invitrogen) in an Eppendorf AF2200 plate reader. The genomic library was prepared using the Nextera XT library preparation kit (Illumina, San Diego, CA) according to the manufacturer’s protocol. The library was quantified using the Kapa Biosystems kit for Illumina on a Roche LightCycler 96 quantitative PCR system. The library was sequenced with 30-fold coverage on the Illumina HiSeq platform using a 250-bp paired-end protocol, producing 1,541,863 reads. Reads were adapter trimmed using Trimmomatic v. 0.30, with a sliding window quality score cutoff value of Q15 (9). FastQC v. 0.11.8 was used to check the quality of the validated reads (10). Reads were *de novo* assembled into 298 contigs using SPAdes v. 3.14 (11). The quality of the assembly was evaluated with Quast v. 5.0.2 (12). Taxonomic assignment of the reads was performed using Kraken v. 2.0.8 (13), and the RAST algorithm v. 2.0 (14–16) was used for genome annotation.

The assembled draft genome had a length of 5,934,315 bp, with a GC content of 65.6%. The largest contig was 162,927 bp long, the *L*_50_ value was 46, and the *N*_50_ value was 40,009 bp. Most of the reads were classified into the genus *Mycobacterium*. The draft genome contained 6,113 unique predicted genes.

Phylogenomic analysis based on the multiple alignment with MUSCLE (17) of the concatenated sequences (53 kbp in length) of 52 single copy genes (18) against the concatenated sequences of the same genes extracted from other known *Mycobacterium* genomes and the construction of a neighbor-joining tree using the Tamura-Nei distance model (19) and 1,000 bootstrap replications showed highest similarity with the gene sequences of Mycobacterium pseudoshottsii (99.2%). Default parameters were used for all software unless otherwise specified.

Our results report the genome sequence of *M. pseudoshottsii* strain AR isolated from meagre, which may provide appropriate prevention and control strategies for Mediterranean aquaculture.

### Data availability.

The whole-genome shotgun project of *M. pseudoshottsii* has been deposited at DDBJ/ENA/GenBank under the accession number JABXJI000000000, BioProject number PRJNA640258, and BioSample number SAMN15312312. Raw sequencing reads were deposited to the Sequence Read Archive under accession number SRP267781. The version described in this paper is version JABXJI000000000.1.

## References

[1] Roo J, Hernández-Cruz CM, Borrero C, Schuchardt D, Fernández-Palacios H 2010 Effect of larval density and feeding sequence on meagre (*Argyrosomus regius*; Asso, 1801) larval rearing. Aquaculture 302:82–88. doi:10.1016/j.aquaculture.2010.02.015.

[2] Tsertou MI, Smyrli M, Kokkari C, Antonopoulou E, Katharios P 2018 The aetiology of systemic granulomatosis in meagre (*Argyrosomus regius*): the “*Nocardia*” hypothesis. Aquaculture Rep 12:5–11. doi:10.1016/j.aqrep.2018.08.002.

[3] Elkesh A, Kantham KPL, Shinn AP, Crumlish M, Richards RH 2013 Systemic nocardiosis in a Mediterranean population of cultured meagre, *Argyrosomus regius* Asso (Perciformes: Sciaenidae). J Fish Dis 36:141–149. doi:10.1111/jfd.12015. 23094711

[4] Avsever ML, Çavuçoʇlu C, Günen MZ, Yazicioʇlu Ö, Eskiizmirliler S, Didinen BI, Tunaligil S, Erdal G, Özden M 2014 The first report of *Mycobacterium marinum* isolated from cultured meagre, *Argyrosomus regius*. Bull Eur Assoc Fish Pathol 34:124–129.

[5] Timur G, Ürkü Ç, Çanak Ö, Erköse Genç G, Erturan Z 2015 Systemic mycobacteriosis caused by mycobacterium marinum in farmed meagre (*Argyrosomus regius*), in Turkey. Isr J Aquac 67:1162.

[6] Rhodes M, Kator H, Kaattari I, Gauthier D, Vogelbein W, Ottinger C 2004 Isolation and characterization of mycobacteria from striped bass *Morone saxatilis* from the Chesapeake Bay. Dis Aquat Organ 61:41–51. doi:10.3354/dao061041. 15584409

[7] Middlebrook G, Cohn ML 1958 Bacteriology of tuberculosis: laboratory methods. Am J Public Health Nations Health 48:844–853. doi:10.2105/ajph.48.7.844. 13545447 PMC1551698

[8] Haught C, Wilkinson DL, Zgafas K, Harrison RG 1994 A method to insert a DNA fragment into a double-stranded plasmid. Biotechniques 16:46–48. 8136137

[9] Bolger AM, Lohse M, Usadel B 2014 Trimmomatic: a flexible trimmer for Illumina sequence data. Bioinformatics 30:2114–2120. doi:10.1093/bioinformatics/btu170. 24695404 PMC4103590

[10] Simon A 2010 FastQC: a quality control tool for high throughput sequence data. Babraham Bioinformatics, Cambridge, United Kingdom.

[11] Bankevich A, Nurk S, Antipov D, Gurevich AA, Dvorkin M, Kulikov AS, Lesin VM, Nikolenko SI, Pham S, Prjibelski AD, Pyshkin AV, Sirotkin AV, Vyahhi N, Tesler G, Alekseyev MA, Pevzner PA 2012 SPAdes: a new genome assembly algorithm and its applications to single-cell sequencing. J Comput Biol 19:455–477. doi:10.1089/cmb.2012.0021. 22506599 PMC3342519

[12] Gurevich A, Saveliev V, Vyahhi N, Tesler G 2013 QUAST: quality assessment tool for genome assemblies. Bioinformatics 29:1072–1075. doi:10.1093/bioinformatics/btt086. 23422339 PMC3624806

[13] Wood DE, Salzberg SL 2014 Kraken: ultrafast metagenomic sequence classification using exact alignments. Genome Biol 15:R46. doi:10.1186/gb-2014-15-3-r46. 24580807 PMC4053813

[14] Aziz RK, Bartels D, Best AA, DeJongh M, Disz T, Edwards RA, Formsma K, Gerdes S, Glass EM, Kubal M, Meyer F, Olsen GJ, Olson R, Osterman AL, Overbeek RA, McNeil LK, Paarmann D, Paczian T, Parrello B, Pusch GD, Reich C, Stevens R, Vassieva O, Vonstein V, Wilke A, Zagnitko O 2008 The RAST Server: rapid annotations using subsystems technology. BMC Genomics 9:75. doi:10.1186/1471-2164-9-75. 18261238 PMC2265698

[15] Overbeek R, Olson R, Pusch GD, Olsen GJ, Davis JJ, Disz T, Edwards RA, Gerdes S, Parrello B, Shukla M, Vonstein V, Wattam AR, Xia F, Stevens R 2014 The SEED and the Rapid Annotation of microbial genomes using Subsystems Technology (RAST). Nucleic Acids Res 42:D206–D214. doi:10.1093/nar/gkt1226. 24293654 PMC3965101

[16] Brettin T, Davis JJ, Disz T, Edwards RA, Gerdes S, Olsen GJ, Olson R, Overbeek R, Parrello B, Pusch GD, Shukla M, Thomason JA, Stevens R, Vonstein V, Wattam AR, Xia F 2015 RASTtk: a modular and extensible implementation of the RAST algorithm for building custom annotation pipelines and annotating batches of genomes. Sci Rep 5:8365. doi:10.1038/srep08365. 25666585 PMC4322359

[17] Edgar RC 2004 MUSCLE: multiple sequence alignment with high accuracy and high throughput. Nucleic Acids Res 32:1792–1797. doi:10.1093/nar/gkh340. 15034147 PMC390337

[18] Eloe-Fadrosh EA, Paez-Espino D, Jarett J, Dunfield PF, Hedlund BP, Dekas AE, Grasby SE, Brady AL, Dong H, Briggs BR, Li W-J, Goudeau D, Malmstrom R, Pati A, Pett-Ridge J, Rubin EM, Woyke T, Kyrpides NC, Ivanova NN 2016 Global metagenomic survey reveals a new bacterial candidate phylum in geothermal springs. Nat Commun 7:10476. doi:10.1038/ncomms10476. 26814032 PMC4737851

[19] Tamura K, Nei M 1993 Estimation of the number of nucleotide substitutions in the control region of mitochondrial DNA in humans and chimpanzees. Mol Biol Evol 10:512–526. doi:10.1093/oxfordjournals.molbev.a040023. 8336541

